# Expanding the Medicinal Chemistry Toolbox for PET Imaging: Aliphatic ^11^C/^18^F‑Trifluoromethylation as a Late-Stage Strategy

**DOI:** 10.1021/cbmi.6c00096

**Published:** 2026-05-19

**Authors:** Xin Zhou, Yinlong Li, Steven H. Liang

**Affiliations:** † Department of Radiology and Imaging Sciences, 1371 Emory University, 1364 Clifton Road, Atlanta, Georgia 30322, United States; ‡ Wallace H. Coulter Department of Biomedical Engineering, Georgia Institute of Technology and Emory University, Atlanta, Georgia 30332, United States

Positron emission tomography (PET) imaging occupies a unique position at the interface of chemistry, biology, and clinical medicine.^1^ Its ability to noninvasively quantify biochemical processes *in vivo* has made PET indispensable in neuroscience, oncology, and drug development.^2–4^ In practice, most clinically used PET radiotracers are derived from a small set of radiochemical transformations, dominated by ^11^C-methylation and nucleophilic ^18^F-fluorination of simple alkyl or aryl precursors.^5–10^ Contemporary drug discovery frequently employs fluorinated motifs, especially CF_3_ groups to optimize pharmacokinetics and binding properties. (Figure 1A)^11–13^ CF_3_ substitution has been exploited to block oxidative metabolism, modulate p*K*_a_ and conformational preferences, and improve membrane permeability and target residence time.^14,15^ Yet many CF_3_-containing drugs and tool compounds remain “PET-inaccessible” because their structural features cannot be readily radiolabeled without extensive scaffold redesign.^16^ The lack of general strategies for forming aliphatic C­(sp^3^)–[^11^C]/[^18^F]­CF_3_ bonds has therefore imposed a structural bias on PET tracer discovery, often favoring radiochemical feasibility over biological relevance.^17^ Aliphatic [^18^F]­CF_3_ groups are conceptually attractive as metabolically robust surrogates for simpler [^18^F]­alkyl motifs.^18,19,16^ Prior approaches including nucleophilic substitution of difluoromethylene precursors or multistep organometallic strategies have suffered from narrow scope, low molar activity, and/or impractical precursor synthesis.^20–22^ In a recent *Science* article, Liu, Pike and co-workers reported a general solution to this challenge by merging copper-mediated radical chemistry with practical radiochemistry.^23^ The authors introduce two complementary pathways for aliphatic radiotrifluoromethylation: halogen atom abstract of alkyl iodides and photoredox-catalyzed decarboxylative trifluoromethylation of carboxylic acids. (Figure 1B) Both approaches rely on radiolabeled fluoroform readily accessible in either containing one of the carbon-11 or fluorine-18 atom as a unified CF_3_ source. It allows the same molecular scaffold to be labeled with either radionuclide, facilitating head-to-head comparison of short-lived ^11^C-tracers and longer-lived ^18^F-analogs without extensive synthetic reoptimization. The reported substrate scope spans primary, secondary, and benzylic centers, tolerates heterocycles and unprotected polar functionality, and accommodates complex, pharmacologically relevant molecules.

**Figure 1:**
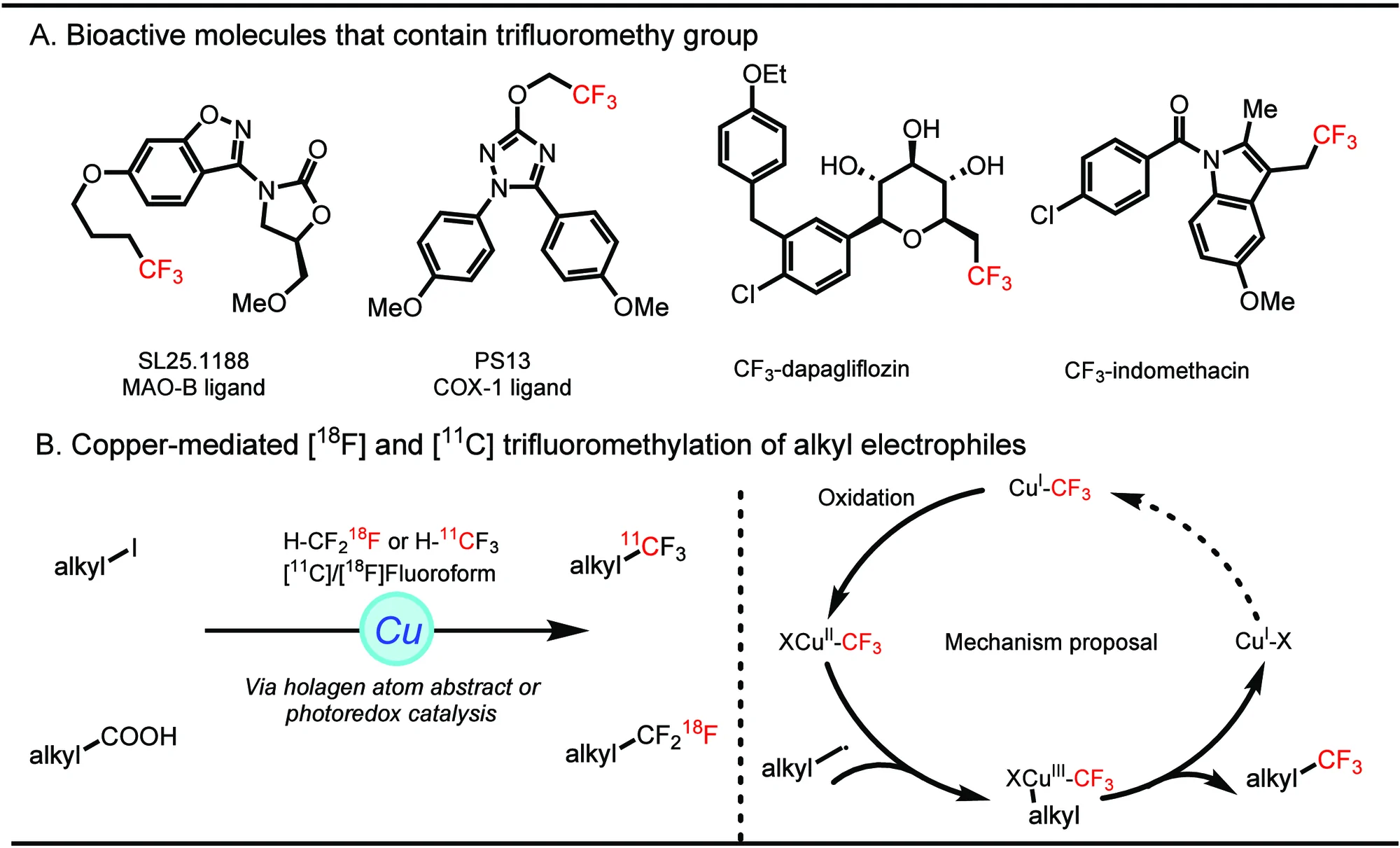
(A) Bioactive molecules with CF_3_ motifs. (B) Copper-mediated ^11^C- and ^18^F- trifluoromethylation and mechanism proposal. The data was adapted from ref 23. Victor W. Pike; Wei Liu et al., *Science*, Copyright 2026 American Association for the Advancement of Science.

From a medicinal chemistry standpoint, the most striking feature of this work is its broad substrate scope and functional group tolerance. Figure 2 illustrates the scope and applicability of the developed trifluoromethylation strategy for the radiosynthesis of [^11^C]­CF_3_-labeled (Figure 2A) and [^18^F]­CF_3_-labeled (Figure 2B) compounds from alkyl iodides. In the presence of CuBr, KO^*t*^Bu and MesN_2_BF_4_, a series of structurally diverse substrates were successfully converted into their corresponding ^11^C-trifluoromethylated products, including late-stage labeling of pharmaceutically relevant molecules such as indomethacin ([^11^C]**4**, 44% yield), tadalafil ([^11^C]**16**, 54% yield), tedizolid ([^11^C]**17**, 53% yield) and dapagliflozin ([^11^C]**18**, 27% yield) derivatives. The reactions proceeded efficiently under mild conditions within 10 min reaction time, delivering the ^11^C-trifluoromethylated products in moderate to good radiochemical yields with high molar activities. The same protocol could also be extended to fluorine-18 chemistry, enabling the formation of [^18^F]­CF_3_-containing analogues from alkyl iodide precursors with comparable efficiency and functional group tolerance, as demonstrated by BAY 59–3074 ([^18^F]**27**, 51% yield, outperforming prior organometallic methods) and the NKCC1 inhibitor IAMA-6, which was radiolabeled either as its methyl ester ([^18^F]**28**, 75% yield) or free acid ([^18^F]**29**, 32% yield). These results demonstrated the generality of this approach and highlighted its utility for rapid late-stage incorporation of PET-relevant trifluoromethyl motifs into complex molecular frameworks. By enabling late-stage installation of CF_3_ groups at aliphatic positions, this methodology allows PET tracers to more accurately reflect the modern structures of drug candidates and clinical leads.

**Figure 2:**
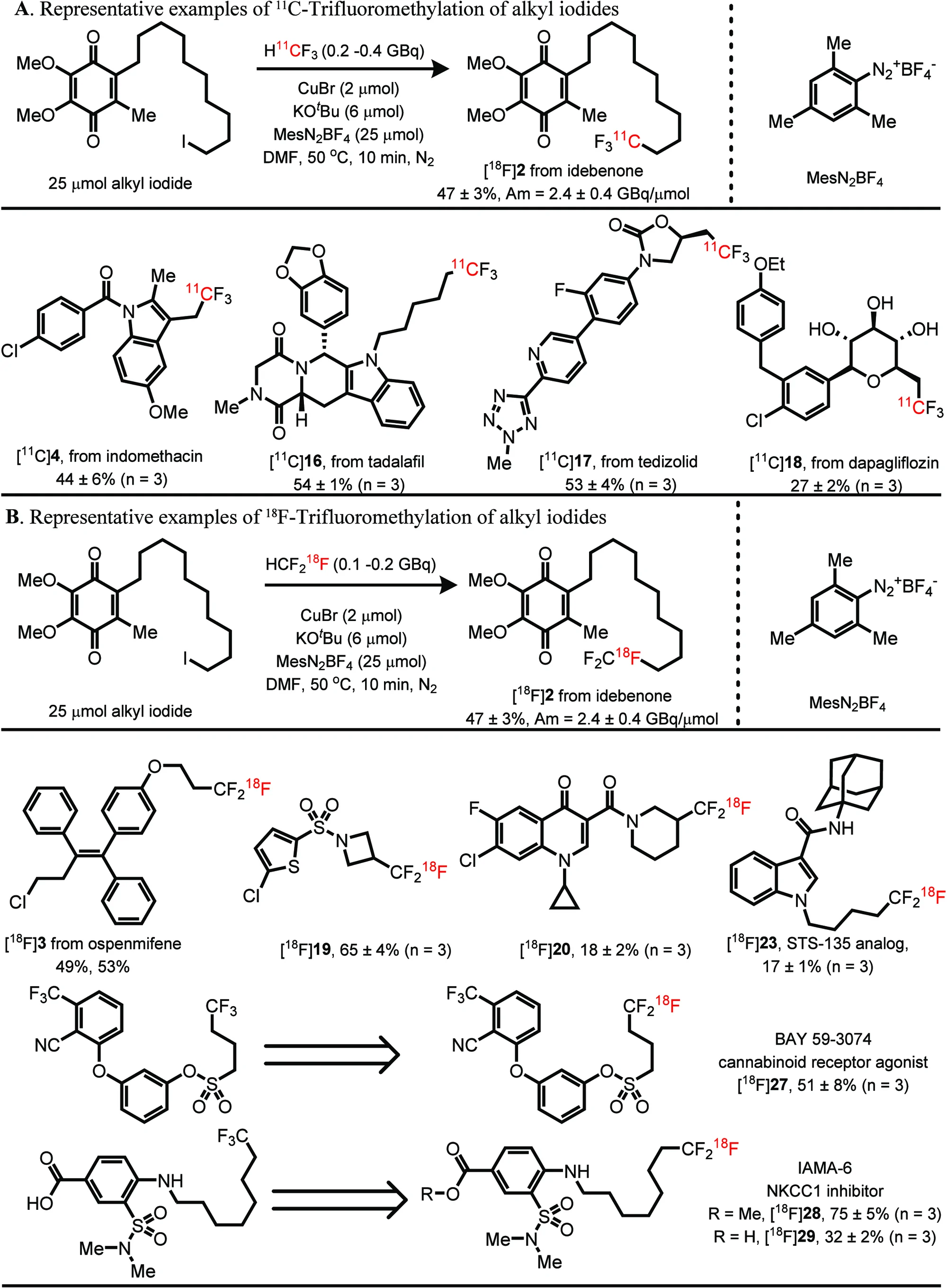
(A) Representative examples of ^11^C-Trifluoromethylation of alkyl iodides. (B) Representative examples of ^18^F-Trifluoromethylation of alkyl iodides. The data was adapted from ref 23. Victor W. Pike; Wei Liu et al., *Science*, Copyright 2026 American Association for the Advancement of Science.

Figure 3 depicts the application of the photocatalytic trifluoromethylation protocol to both carbon-11 and fluoroine-18 radiochemistry using [^11^C]­fluoroform or [^18^F]­fluoroform as the CF_3_ source, respectively. Under the optimized conditions, involving CuBr, KO^t^Bu, Ru­(bpy)_3_Cl_2_·6H_2_O, and blue LED irradiation at room temperature, a range of alkyl iodide substrates were efficiently converted to the corresponding [^11^C]­CF_3_- or [^18^F]­CF_3_-labeled products within 10 min. The methodology enabled direct labeling of heterocycle-containing alkyl chains ([^11^C]**38**, 59% yield) as well as benzylic ([^11^C]**40**, 88% yield; [^11^C]**43**, 17% yield) and aliphatic systems ([^11^C]**44**, 84% yield), affording ^11^C-labeled products in good radiochemical yields with high molar activity. Notably, the same reaction manifold was readily translated to fluorine-18 chemistry and provided [^18^F]­CF_3_-containing analogues in comparable radiochemical yields and reproducibility. A series of primary ([^18^F]**37**, 69%, 73% yield; [^18^F]**39**, 65% yield), secondary ([^18^F]**41**, 43% yield) and tertiary ([^18^F]**42**, 65%) aliphatic acid were successfully converted to [^18^F]­CF_3_-containing compounds. These results highlight the versatility of this unified strategy for rapid incorporation of PET-relevant ^11^C and ^18^F-trifluoromethyl motifs using short-lived radionuclides.

**Figure 3:**
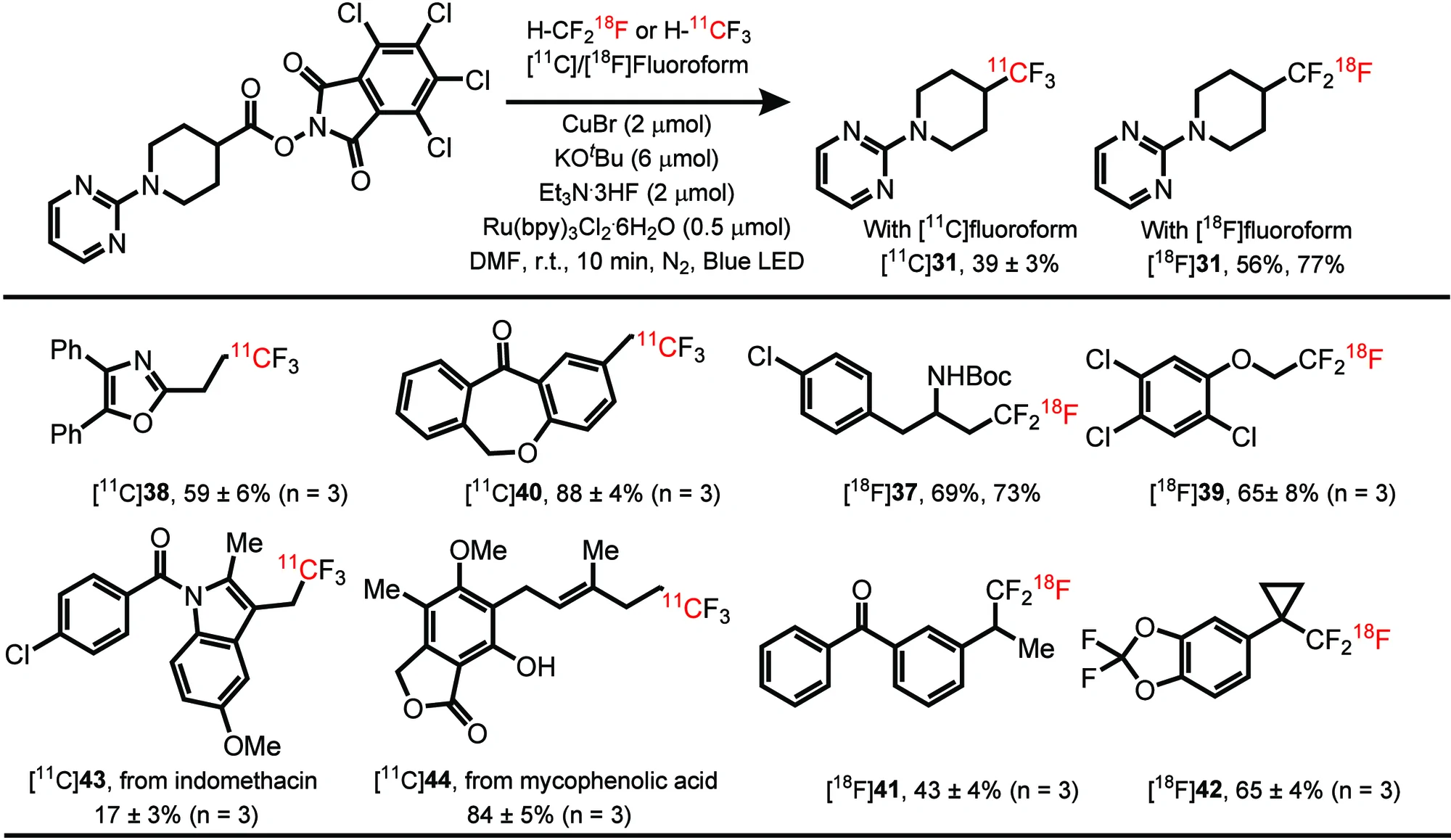
Representative examples of Decarboxylative [^11^C]-and [^18^F]-trifluoromethylation of alkyl carboxylic acids. The data was adapted from ref 23. Victor W. Pike; Wei Liu et al., *Science*, Copyright 2026 American Association for the Advancement of Science.

## Concluding Remarks

The work by Liu, Pike and co-workers reflects a growing trend in PET radiochemistry by embracing radical-based activation and late-stage functionalization. Looking forward, the ability to access matched [^11^C]/[^18^F]­CF_3_ tracers from a single precursor opens new experimental paradigms. For example, rapid same day ^11^C studies could be used for early target engagement assessment, followed by ^18^F tracers for longitudinal or multicenter imaging. Such flexibility aligns well with contemporary translational research models and demonstrates that aliphatic C­(sp^3^)–CF_3_ radiolabeling can be both general and practical. Continued advances in high-activity [^18^F]­fluoroform production, automation, and microfluidic photochemistry are likely to push this methodology to a higher place. Despite its conceptual achievements, several limitations may affect the broader adoption of this methodology. There is further optimization opportunity to increase molar activity of [^18^F]­CF_3_ products derived from fluoroform-based labeling using different generation methods. In addition, the requirement for radiolabeled gaseous fluoroform introduces operational complexity compared with conventional [^18^F]­fluoride chemistry, which mandates a simplified workflow suitable for routine clinical translation. In summary, general aliphatic radiotrifluoromethylation represents a meaningful expansion of the medicinal chemist’s imaging toolbox. By reducing the structural gap between drugs and tracers, this chemistry brings PET imaging closer to its full potential as a translational bridge between molecular design and clinical insight.

## Abbreviations

PET: positron emission tomography
NKCC1: Na–K-2Cl cotransporter 1
